# Th17 and Treg Balance in Children With Obesity and Metabolically Altered Status

**DOI:** 10.3389/fped.2020.591012

**Published:** 2020-11-19

**Authors:** Valeria Calcaterra, Stefania Croce, Federica Vinci, Annalisa De Silvestri, Erika Cordaro, Corrado Regalbuto, Gian Vincenzo Zuccotti, Chiara Mameli, Riccardo Albertini, Maria Antonietta Avanzini

**Affiliations:** ^1^Pediatric and Adolescent Unit, Department of Internal Medicine, University of Pavia, Pavia, Italy; ^2^Department of Pediatrics, Children's Hospital “V. Buzzi”, Milan, Italy; ^3^Cell Factory, Pediatric Hematology Oncology Unit, Immunology and Transplantation Laboratory, Department of Maternal and Children's Health, Fondazione Istituto di Ricovero e Cura a Carattere Scientifico Policlinico S. Matteo, Pavia, Italy; ^4^Pediatric Unit, Department of the Mother and Child Health, Fondazione Istituto di Ricovero e Cura a Carattere Scientifico Policlinico San Matteo, University of Pavia, Pavia, Italy; ^5^Biometry & Clinical Epidemiology, Scientific Direction, Fondazione Istituto di Ricovero e Cura a Carattere Scientifico Policlinico San Matteo, Pavia, Italy; ^6^Department of Biomedical and Clinical Science L. Sacco, University of Milan, Milan, Italy; ^7^Laboratory of Clinical Chemistry, Fondazione Istituto di Ricovero e Cura a Carattere Scientifico Policlinico San Matteo, Pavia, Italy

**Keywords:** obesity, children, Th17, Treg, metabolic status

## Abstract

**Background:** Chronic low-grade inflammation and activation of the immune system are hallmark pathogenic mechanisms involved in metabolic dysfunction and are related to obesity. In particular, the involvement of regulatory and pro-inflammatory lymphocyte subpopulations has been reported in adults. We evaluated the Th17/Treg lymphocyte balance in obese and normal weight children, in relation with their metabolic status.

**Methods:** We enrolled 50 pediatric patients. According to metabolic status, subjects were classified into: metabolically healthy (MH) and metabolically unhealthy (MU) groups. MU phenotype was defined as the presence of at least one of the following risk factors: blood pressure >90th percentile, glycemia>100 mg/dl, HDL cholesterol <40 mg/dl, triglycerides>100 mg/dl (<10 years) or >130 mg/dl (>10 years), impaired insulin sensitivity with HOMA-IR>97.5th percentile. Patient Treg and Th17 profiles were also evaluated.

**Results:** Based on the presence of metabolic and/or cardiovascular pathological parameters, we classified 15 MU (30%) and 35 MH (70%) children; all MU children were obese. Analyzing the correlations between lymphocyte subpopulations and metabolic data, we noted a correlation between Th17 percentage and systolic hypertension (*p* = 0.01, *r* = −0.37); Treg/Th17 ratio and HOMA-IR (*p* = 0.02, *r* = 0.32) and systolic hypertension (*p* = 0.05, *r* = 0.30).

**Conclusion:** Children with obesity have a high risk of developing metabolic and cardiovascular complications. The Th17/Treg lymphocyte balance appears to be involved in glycemic homeostasis and blood pressure control. Careful and early monitoring of the immune system would facilitate new early preventive strategies in pediatric metabolic diseases.

## Introduction

The global obesity epidemic is worrying particularly considering the involvement of children and adolescents (1–5). Metabolic and cardiovascular complications linked to excess weight have been identified in young age groups, with important health and economic consequences (6–11).

Obesity is associated with systemic inflammation and is characterized by the presence of CD4 and CD8 T cell infiltration and altered immune homeostasis (12, 13). Compared to subcutaneous adipose, visceral adipose tissue has higher levels of acute phase proteins (14–17).

Importantly, this tissue turns out to be the main seat of immune cell production, and these cells in turn produce a considerable amount of pro-inflammatory cytokines (18–23), such as TNFα (16, 24), IL-1β (25, 26), IL-6 (16, 24), and interferon (IFN) γ (27–29).

It is largely recognized that chronic low-grade inflammation and activation of the immune system are involved in the pathogenic mechanisms of metabolic dysfunction (30). Data from the literature, both in experimental and human models, show the involvement of regulatory and pro-inflammatory lymphocyte subpopulations, such as T regulatory cells (Treg) and Th17. Th17 and Treg cells have reciprocal developmental pathways and opposing effects, proinflammatory, and immunosuppressive, respectively (30, 31). The factors controlling the Treg/Th17 balance and their reciprocal relationship have not been fully elucidated. However, the balance between these two compartments is central to the pathogenesis of various diseases and conditions including obesity-related complications (30–34). In particular, the role of Th17/Treg cell balance has been reported in the development and progression of insulin resistance (35), type 2 diabetes (36), non-alcoholic fatty liver disease (37–39), and atherosclerosis (40). To the best of our knowledge, no data on this topic have been described in the pediatric population.

The aim of this study was to evaluate the Th17/Treg lymphocyte balance in obese and normal weight children, in relationship with their metabolic status, in order to define its role in the development of metabolic complications.

## Patients and Methods

### Patients

We consecutively enrolled 50 Caucasian children and adolescents (24 M and 26 F; mean age 11.8 ± 3.6 years). These subjects were referred by their general practitioner or primary care pediatrician for auxological evaluation or obesity, between November 2018 and June 2019, to the outpatient clinic of the Pediatric Endocrinology Unit at the Fondazione IRCCS Policlinico S. Matteo. Exclusion criteria included: known secondary obesity conditions, use of any medications, and concomitant chronic or acute illnesses.

Subjects were divided into two groups, according to the World Health Organization criteria: (41)

- subjects with obesity (*n* = 33): BMI ≥97th percentile for the age and sex,
- subjects with normal weight (*n* = 17): BMI <85th percentile for the age and sex.

To assess dietary habits and physical activity, we used two sections of a previously validated questionnaire (42). Each section consisted of: 14 questions on dietary habits (breakfast consumption, daily water assumption, daily number of meals, and consumption of fruit, vegetables, and soft drinks or alcoholic beverages) and 5 questions on physical activity (walking, watching TV, listening, to music, using the computer, reading, practicing a sport, and shopping) with the following response categories: always, often, sometimes, never. The score assigned to each response ranged from 0 to 3, with the maximum score assigned to the healthiest answer and the minimum score to the least healthy response (42).

According to metabolic status, described in the Methods section, the patients were further classified into: metabolically healthy (MH) or metabolically unhealthy (MU).

The study protocol was approved by the Institutional Review Board of the Fondazione IRCCS Policlinico San Matteo (prot. 20190043635), and carried out in accordance with the Helsinki Declaration of 1975, as revised in 2008. All participants' parents or their responsible guardians were asked for and gave their written consent after being informed about the nature of the study.

## Methods

### Physical Examination

The physical examination of the participants included anthropometric evaluation: height, weight, waist circumference, BMI calculation, pubertal stage determination according to Marshall and Tanner (prepubertal characteristics corresponding to Tanner stage 1) (43, 44) and blood pressure (BP) measurement. Height, weight, and waist circumference measurement, were performed as previously reported (45). BMI was calculated as body weight (kilograms) divided by height (meters squared) and waist to height ratio (WHtR) was also considered as a central adiposity index. Pubertal development was classified as: stage 1 = Tanner 1; stage 2 = Tanner stages 2–3; and stage 3 = Tanner stages 4–5. Systolic (SBP) and diastolic (DBP) blood pressures were measured twice with the patient sitting quietly; the second BP measurement was used for the analysis.

### Biochemical Parameters

Blood samples were drawn in the morning, after an overnight fast. Metabolic blood assays included: glucose, insulin, total cholesterol, HDL-cholesterol, triglycerides (TGs), AST, ALT, GGT, PCR. Insulin resistance was calculated with the homeostasis model assessment for insulin resistance (HOMA-IR) formula. Plasma glucose was measured using the hexokinase-G-6-phosphate dehydrogenase method (Siemens Healthcare Diagnostics, UK) with a chemistry analyzer (Advia XPT, Siemens). Total cholesterol was determined with an enzymatic method (Advia XPT, Siemens Healthcare Diagnostics, UK). HDL cholesterol was measured with the selective detergent method, followed by enzymatic reactions (Siemens Healthcare Diagnostics). TG concentrations were measured with the glycerol phosphatase oxidase method (Siemens Healtcare Diagnostics, UK). Serum insulin was measured with a solid-phase, two-site chemiluminescent immunometric assay with an immunochemistry analyzer (Immulite 2000, Siemens Healtcare Diagnostics, UK). AST, ALT, and GGT were measured with a chemistry analyzer (Advia XPT, Siemens Healthcare) equipped with dedicated reagents; the method for the transaminase assay is based on NADH monitoring by ultraviolet (UV) detection without addition of P-5′-P. The GGT assay method is based on the transfer of the gamma-glutamyl group from L-gamma-glutamyl-3-carboxy-4-nitroaniline to the glycilglycine acceptor, to yield 3-carboxy-4-nitroaniline, which is measured. PCR was measured with clinical chemistry methods using the Advia XPT (Siemens Healthcare).

### MH and MU Definition

To define MH subjects, we applied the following criteria: SBP and DBP <90th percentile by gender, age and height percentile, glycemia <100 mg/dl, HDL-cholesterol >40 mg/dl, triglycerides <100 mg/dl (children <10 years) or <130 mg/dl (children >10 years), normal insulin sensitivity (ISI) with HOMA-IR ≤97.5 percentile for age and sex (46–48).

The MU phenotype was defined as the presence of at least one of the following risk factors: SBP or DBP >90th percentile, glycemia >100 mg/dl, HDL cholesterol <40 mg/dl, triglycerides >100 mg/dl (children <10 years) or >130 mg/dl (children >10 years), impaired insulin sensitivity (ISI) with HOMA-IR exceeding the 97.5th percentile for age and sex (48).

The criteria to define metabolic status were based on a consensus of the literature (≥80% agreement) (46). As markers of glycol metabolic derangement, a fasting blood glucose, and/or ISI was used. Impaired fasting glucose is rare in childhood and insulin resistance precedes glucose abnormalities and plays an important role in the transition from normal glucose tolerance to impaired glucose tolerance. Although the euglycemic-hyperinsulinemic clamp procedure is the gold standard method for the determination of insulin sensitivity, it is an impractical test in children as it is invasive and time intensive. For these reasons, we used HOMA-IR as a surrogate marker of insulin resistance/sensitivity (49, 50).

### Peripheral Blood Mononuclear Cell Isolation

Peripheral blood mononuclear cells (PBMCs) were obtained by gradient density separation (Lympholyte, Cederlane). After two washes with saline solution (0.9% NaCl), viable PBMCs were cryopreserved (1% DMSO) in liquid nitrogen until analyses were performed.

After, thawing at 37°C, viable PBMCs were counted with trypan blue. Cells were suspended at a density of 1 × 10^6^ cells/ml in RPMI supplemented with 10% heat-inactivated fetal calf serum (Euroclone) for cell surface and intracellular staining.

### Treg and Th17 Analyses

PBMCs were stained for Treg cell surface markers following standard procedures. In brief, after an incubation at +4°C for 30 min with antibodies specific for cell surface antigens (PC7 labeled anti-CD4, FITC labeled anti-CD127, APC labeled anti-CD25; Beckman Coulter), the cells were treated with fixation/permeabilization buffer (eBioscience) at +4°C for 40 min. PBMCs were then washed three times with permeabilization buffer to allow intracellular staining with PE-specific for forkhead box P3 transcription factor (FoxP3) antibody (eBioscience) at +4°C for 30 min.

In order to detect Th17, PBMCs were stimulated with phorbol 12-myristate l3-acetate (PMA) and ionomycin for 4 h at 37°C in the presence of brefeldin A. After one wash, cells were incubated for 30 min at +4°C with FITC labeled anti-CD3 and APC labeled anti-CD4 (Beckman Coulter). After the permeabilization procedure with fixation/permeabilization buffer (eBioscience) at +4°C for 40 min, PBMCs were washed three times with permeabilization buffer to allow intracellular staining with PE anti-IL17 (BD) at +4°C for 30 min.

For both staining procedures, appropriate isotype-matched controls were used. Acquisition and analysis of cell populations were performed by direct immunofluorescence with a Navios flow cytometer (Beckman Coulter, Milan, Italy). Typically, 100,000 events were acquired.

**Figure 1:**
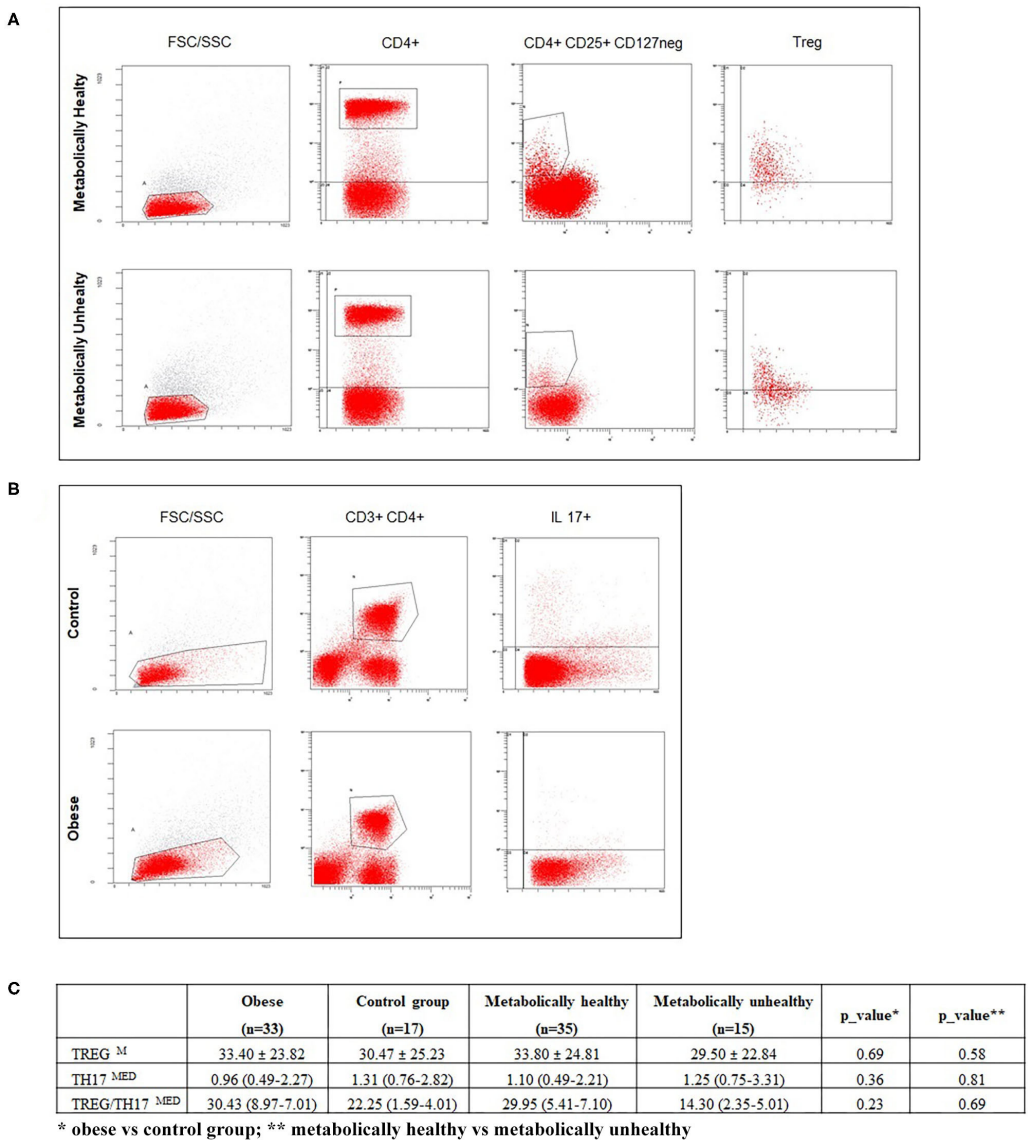
**(A,B)** respectively, show representative flow cytometry analyses of Treg and Th17 subpopulations. **(C)** reports percentages of Treg, Th17 cells, and the Treg/Th17 ratio in enrolled subjects, according to BMI and metabolic status. Data are expressed as the mean ± SD (M) or median and IQR (MED). A *p* ≤ 0.05 was considered significant.

Tregs were defined as CD4+ CD127 neg CD25+ cells expressing FoxP3. Th17 were defined as CD4+ cells expressing intracellular IL-17. Representative flow cytometry analyses of Treg and Th17 cells, with gating algorithms, are reported in Figure 1.

### Statistical Analysis

All analyses were performed using Stata 16 (StataCorp, College Station, TX, USA). Data were described with the mean, standard deviation (SD), median and 25th−75th percentiles if continuous and as counts and percent if categorical. Comparisons of quantitative variables between groups were made with the independent *t*-test or Mann-Whitney test. The association of categorical variables was assessed with the Fisher's exact test. For the purpose of this analysis, biomarkers were dichotomized at the local laboratory cut-off for normality. All tests were 2-sided. A *p* < 0.05 was considered statistically significant. Non-parametric correlations between continuous variables were assessed with the Spearman *R*-test. The magnitude of an effect size for correlation coefficients was evaluated as follows, as described by Cohen: small for correlation coefficients on the order of 0.1, medium for those on the order of 0.3, and large for those on the order of 0.5 (51).

## Results

### Clinical and Metabolic Parameters

The characteristics of enrolled obese children and normal weight subjects are reported in Table 1. No differences in age or sex were revealed between the groups. Children with obesity had average BMIs and HOMA-IR values, but significantly higher values when compared to normal weight subjects (*p* < 0.001). No other significant differences in mean values of metabolic parameters were noted. Based on the presence of metabolic and/or cardiovascular pathological parameters, 15 MU (30%) and 35 MH (70%) children were identified.

**Table 1:** Clinical and biochemical characteristics of enrolled subjects, according to BMI and metabolic status.

	**Obesity (*n =* 33)**	**Normal weight (*n =* 17)**	**Metabolically healthy (*n =* 35)**	**Metabolically unhealthy (*n =* 15)**	*P*-value^*^	*P*-value^**^
Age^M^	11.43 ± 3.53	12.73 ± 3.68	11.52 ± 3.44	12.7 ± 3.93	0.22	0.29
BMI^M^	24.86 ± 4.03	19.02 ± 3.95	21.16 ± 3.98	26.88 ± 4.39	<0.001	<0.001
Pubertal stage Tanner 1 Tanner 2 Tanner 3	13 (39.39) 13 (39.39) 7 (21.21)	6 (35.29) 4 (23.53) 7 (41.18)	15 (42.86) 11 (31.43) 9 (25.71)	4 (26.67) 6 (40) 5 (33.33)	0.28	0.55
Physical activity	8 (2.51)	9.82 (2.35)	9.37 (2.21)	6.87 (2.61)	0.01	<0.001
Diet	31.64 (4.71)	33.06 (4.53)	32.74 (4.41)	30.67 (5.04)	0.31	0.15
BG (mg/dl) ^M^	73.76 ± 9.89	77.13 ± 13.48	74.88 ± 11.48	73.6 ± 9.13	0.42	0.71
HbA1c (%)^M^	5.22 ± 0.27	5.4 ± 0.23	5.22 ± 0.28	5.33 ± 0.25	0.09	0.22
TC (mg/dl)^M^	150.48 ± 26.45	128 ± 21.21	147.55 ± 17.02	151.4 ± 36.02	0.24	0.67
HDL-c (mg/dl)^M^	47.48 ± 9.86	49.5 ± 2.12	51.85 ± 5.58	41.93 ± 10.98	0.77	0.001
TGs (mg/dl)^M^	71.88 ± 33.36	63 ± 39.6	60.7 ± 19.89	85.6 ± 41.84	0.71	0.029
SBP (mmHg)^M^	107.73 ± 10.9	104.25 ± 10.97	103.7 ± 10.05	113 ± 10.14	0.35	0.005
DBP (mmHg)^M^	66.36 ± 7.1	67.75 ± 7	65.1 ± 6.51	70 ± 7.07	0.56	0.02
INSULIN (ml/UL)^MED^	8.8 (5.3–15.7)	6 (4.4–9.6)	6.4 (4.8–9.6)	13 (6.2–19)	0.56	0.32
HOMA-IR^MED^	1.70 (0.9–2)	0.9 (0.6–1.1)	0.94 (0–1.7)	2.21 (1.1–3)	<0.01	0.07
AST (mU/ml)^M^	24.78 ± 9.14	23 ± 4.24	22.63 ± 2.79	27.27 ± 12.8	0.78	0.13
ALT (mU/ml)^MED^	16 (13–24.5)	14.5 (10–19)	16 (13–19)	20 (13–34)	0.61	0.25
GGT (mU/ml)^MED^	9 (7.5–14)	8 (7–13)	9 (7.5–13)	9 (8–14)	0.62	0.40
CRP (mg/dl)^MED^	0.26 (0.05–0.61)	0.5 (0.5–0.5)	0.22 (0.09–0.5)	0.34 (0.04–1.08)	0.71	0.68

*Data are expressed as the mean ± SD (M) or median and IQR (MED)*.

* *obese vs. normal weight*;

** *Metabolically healthy vs. metabolically unhealthy. AST, aspartate transaminase; ALT, alanine aminotransferase; BMI, body mass index; DBP, diastolic blood pressure; BG, blood glucose; GGT, gamma glutamyl transpeptidase; HDL-c, high-density lipoprotein-cholesterol; HOMA-IR, homeostatic model assessment- insulin resistance; CRP, C-reactive protein; SBP, systolic blood pressure; TC, total cholesterol; TGs, triglycerides*.

In detail, pathological cholesterol-HDL values were noted in 10 patients (30.3%), hypertriglyceridemia in four children (12.1%), systolic hypertension pressure in two (6.1%) and diastolic pressure in two (6.1%) subjects. All patients that presented with pathological parameters were obese. As reported in Table 1, BMI (*p* < 0.001), TG (*p* = 0.029), SBP (*p* = 0.005), and DBP (*p* = 0.02) were significantly higher in subjects with dysmetabolism, while HDL-cholesterol (*p* = 0.001) was significantly lower than that in metabolically normal subjects. Furthermore, only borderline significant (*p* = 0.07) HOMA was higher in the MH group when compared with the MU group.

### Immunological Data

Regarding immunological parameters, the percentages of lymphocyte subsets tested in normal weight and obese children and in metabolically normal and metabolically altered subjects are shown in Figure 1. We observed a decreasing circulating Th17 trend in children with obesity compared with normal weight children and an increasing Treg percentage trend in MH compared with MU children, without reaching significance, Figure 1.

**Figure 2:**
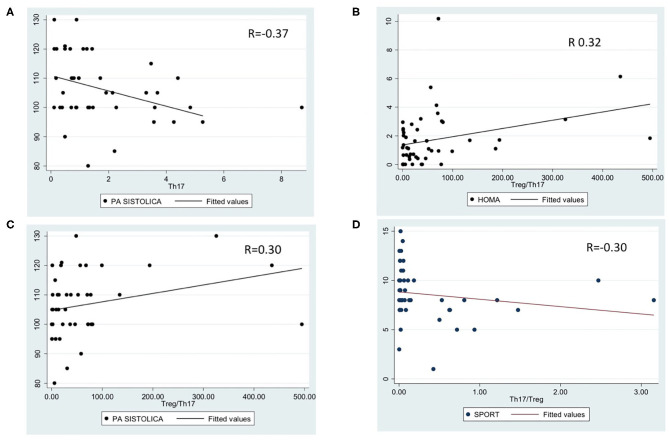
Significant correlations between lymphocyte subpopulations and metabolic data. **(A)**: correlation between Th17 percentage and systolic hypertension (*p* = 0.01, *r* = −0.3769); **(B)**: correlation between Treg/Th17 ratio and HOMA-IR (*p* = 0.02, *r* = 0.32); **(C)**: correlation between Treg/Th17 ratio and systolic hypertension (*p* = 0.05, *r* = 0.30); **(D)**: correlation between Treg/Th17 ratio and physical activity (*p* = 0.04, *r* = −0.30).

Analyzing the correlations between lymphocyte subpopulations and clinical or metabolic data, we noted a significant correlation between Th17 percentage and systolic hypertension (*p* = 0.01, *r* = −0.37, Figure 2A); Treg/Th17 ratio and HOMA-IR (*p* = 0.02, *r* = 0.32, Figure 2B), systolic hypertension (*p* = 0.05, *r* = 0.30, Figure 2C) and physical activity level (*p* = 0.04, *r* = −0.30, Figure 2D). No other significant differences were detected.

## Discussion

Childhood obesity has reached the point where it should be considered a public health emergency (1–5). As confirmed by our results, a pathological BMI is the main risk factor for the development of metabolic and cardiovascular complications. Obese children should be evaluated for insulin resistance, dyslipidemia, and hypertension, which represent the most common early risk factors in children (6–11).

Obesity is associated with low-grade systemic inflammation; this state is well-defined by altered circulating levels of cytokines and acute phase reactants (11). During the development of an overweight or obese state, the adipose tissue undergoes morphological and secretive changes leading to a Th1 bias and a chronic state of inflammation (1, 11–17) that is involved in the development of numerous complications, such as insulin-resistance, hypertension, and dyslipidemia (18, 20, 21, 24).

Various T cell subpopulations regulate the cytokine balance of the body and secrete inflammatory factors, suggesting a link between T cell regulation and the pathogenesis of obesity related diseases. In particular, an imbalance in Th17 and Treg lymphocyte subsets has been proposed to play a role in obesity-dependent inflammation and related complications, including type 2 diabetes (T2DM) and its precursor IR (20, 24, 27, 52, 53) and early atherosclerosis process (18, 40, 54).

Even though we observed increasing or decreasing trends in Th17 and Treg lymphocytes, without reaching statistical significance, we noted a significant correlation between the Th17 and Treg distribution and the presence of IR and hypertension. These data support the influence that T cell imbalance has on systemic inflammation and dysmetabolism (11–17, 34, 55–60) and suggest a role for T cell subsets in obesity-related complications (27–39, 52–60). It has been shown that adipose tissue is a true endocrine organ able to produce and to release numerous inflammatory mediators, which play an important role in energetic homeostasis, in modulation of inflammation and the immunological response (52–60). In particular, IL-6 and TNFα seem to directly interfere with the normal transmission of insulin signals, promoting the onset of insulin resistance and later the onset of type 2 diabetes (61–64). The imbalance of lymphocyte subpopulations both in the presence of hypertension and IR confims that the two conditions may be related to each other and that inflammation is implicated in early angiogenetic processes (18, 34, 40, 54). In our study, pubertal changes may have influenced insulin sensitivity values. Moreover, considering that pathological HOMA-IR for age, sex, and pubertal stage, is accepted as a good marker of IR and IR confers risks for dyslipidemia and hypertension at a relatively early age in pediatrics (65), the role of immune system monitoring in prevention should be emphasized.

The correlation between Treg/Th17 ratio and physical activity levels confirms the importance of exercise on immune cell function, strengthening the crucial role of preventive programs.

We acknowledge that this study has some limitations, including the relatively small patient sample size that may have influenced the statistical power of the results. However, this is one of the few studies in the scientific literature evaluating this immunologic aspect in the pediatric age. Secondly, our results show an association, but do not demonstrate a direct cause-and-effect relationship, between Th17 and Treg imbalance and obesity related complications. Additionally, we only considered the role of Th17 and Tregs in metabolic disorders, confident that their cytokine expression would be useful to define the pathogenic mechanism in an inflammatory metabolically altered state. Finally, the role of fat distribution in cardiometabolic risk has been reported (66) and fat distribution at different body locations has been associated with changes in different types of cardiovascular risk biomarkers (67–69). In our patients, body composition was not recorded and the influence of fat phenotype on cardiometabolic and immunological profiles cannot be excluded.

Further studies are necessary to confirm our results and to define the role of inflammation in the complications related to obesity and the role regulatory T cells have in protection or predisposition, also considering different degrees of ponderal excess and fat distribution.

In conclusion, obese pediatric patients have a high risk of developing metabolic and cardiovascular diseases. Adipose tissue appears to be involved in T cell regulation of metabolic and tissue inflammatory responses. Careful and early monitoring of the immune system would offer new early preventive strategies in pediatric metabolic diseases.

## Data Availability Statement

The datasets generated for this study are available on request to the corresponding author.

## Ethics Statement

The studies involving human participants were reviewed and approved by Fondazione IRCCS Policlinico San Matteo (prot. 20190043635). Written informed consent to participate in this study was provided by the participants' legal guardian/next of kin.

## Author Contributions

VC and MA designed the experiments, wrote, and supervised the writing of the manuscript. SC, FV, EC, and CR performed the experiments and wrote the manuscript. GVZ and CM supervised the writing of the manuscript. AD performed statistical analyses. RA performed the biochemical evaluations and wrote the manuscript. All of the authors have read and approved the manuscript.

## Conflict of Interest

The authors declare that the research was conducted in the absence of any commercial or financial relationships that could be construed as a potential conflict of interest.
